# The mechanism and clinical significance of FKBP5 gene DNA methylation in various psychiatric, metabolic and tumor-related diseases

**DOI:** 10.3389/fgene.2026.1734673

**Published:** 2026-01-26

**Authors:** Changliang Wang, Zhixiu Xia

**Affiliations:** 1 Judicial Authentication Center, The People’s Procuratorate of Liaoning Province, Collaborative Laboratory of Intelligentized Forensic Science (CLIFS), Shenyang, China; 2 Department of General Surgery, Shengjing Hospital of China Medical University, Shenyang, China

**Keywords:** biomarker, DNA methylation, epigenetics, FKBP5, targeted therapy

## Abstract

The FK506 Binding Protein 5 (FKBP5) gene encodes a protein that binds to the immunosuppressive agent FK506. FKBP5 expression is regulated by genetic variation and epigenetic mechanisms, including DNA methylation (DNAm). This gene regulates the glucocorticoid receptor (GR), and aberrant FKBP5 methylation is associated with psychiatric and metabolic disorders. Recent evidence also indicates that FKBP5 methylation significantly influences malignant tumors. The methylation status of FKBP5 not only modulates its own expression but also contributes to disease pathogenesis by regulating downstream signaling pathways. Despite extensive research on FKBP5 in individual disease contexts, a critical gap remains in understanding how its DNAm serves as a unifying epigenetic mechanism across psychiatric, metabolic, and neoplastic disorders. Existing reviews often focus on single disease domains or on genetic and protein-level regulation, lacking a systematic, horizontal integration analysis centered on DNAm—a dynamic and reversible modification. This review aims to fill this gap by proposing a coherent “epigenetic regulatory framework” that elucidates how tissue-and site-specific FKBP5 DNAm patterns, through modulating glucocorticoid (GC) signaling, stress responses, and inflammatory pathways (e.g., NF-κB), contribute to divergent pathological outcomes. By integrating evidence from disparate fields, this review summarizes the role of FKBP5 DNAm in disease biology, its functions across various disorders, and its potential as a biomarker and therapeutic target, aiming to provide a theoretical foundation and strategic insights for disease diagnosis and treatment.

## 1 Introduction

Epigenetics encompasses heritable changes in gene expression that occur without alterations to the DNA sequence itself (Ma et al., 2025; Akano et al., 2025). These changes, which include DNAm, can be stably maintained through cell divisions and are crucial for regulating normal development and cellular responses. DNAm involves the addition of a methyl group to cytosine bases within CpG dinucleotides, leading to modulation of transcriptional activity and chromatin architecture. Beyond its physiological roles, dysregulated DNAm is increasingly implicated in the pathogenesis of numerous diseases (Aslam and Ageed, 2024; Antrobus et al., 2025; van Vliet et al., 2024; Tan et al., 2024), with alterations exhibiting both tissue specificity and dynamic regulation over time (Dabin et al., 2020). This positions epigenetic mechanisms, particularly DNAm, as central players in precision medicine, offering novel avenues for diagnosis and therapy (Pinho and Maga, 2021; Zima et al., 2022).

Environmental exposures, such as tobacco smoke and alcohol, are potent modifiers of the epigenome through gene-environment interactions (Akhabir et al., 2022; Dragic et al., 2022; Stols-Gonçalves et al., 2024; White et al., 2024; Fadadu et al., 2025). For instance, studies using epigenome-wide approaches have consistently identified demethylation at specific CpG sites within the FKBP5 gene in peripheral blood mononuclear (PBM) cells following such exposures (Dogan et al., 2016). FKBP5, a critical regulator of the hypothalamic-pituitary-adrenal (HPA) axis stress response, itself undergoes environmentally driven DNAm changes and appears to facilitate broader epigenetic reprogramming. Notably, stress-related methylation abnormalities in FKBP5 have been observed in brain regions like the prefrontal cortex (PFC) and hippocampus, contributing to the pathophysiology of neuropsychiatric conditions such as alcohol use disorder (Gatta et al., 2021). Similarly, environmental factors can drive tissue-specific methylation changes in oncogenes, highlighting a pervasive role for epigenetics in disease (Saville et al., 2025).

**FIGURE 1:**
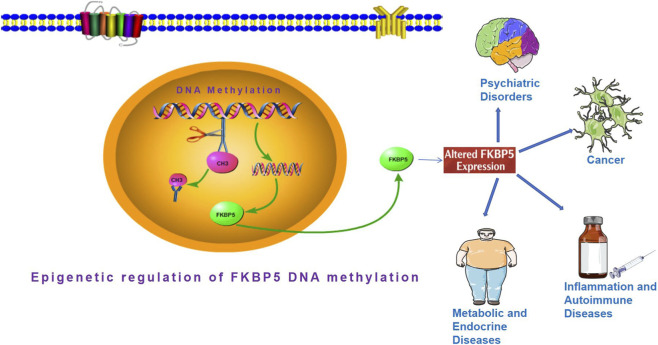
Schematic diagram of the scientific hypothesis of epigenetic regulation of FKBP5 DNA methylation.

Altered FKBP5 DNA methylation (FKBP5-DNAm) is associated with a spectrum of disorders, including stress-related psychiatric conditions, metabolic diseases, and various cancers (Klengel et al., 2013; Rysz et al., 2022). These findings position FKBP5 as a promising biomarker and a novel therapeutic target, offering new avenues for disease prevention and treatment. However, while substantial research has been conducted on FKBP5 in isolated disease contexts, previous studies have predominantly focused on single disease domains-such as depression, post-traumatic stress disorder (PTSD), diabetes, or specific cancers-or have emphasized genetic variants and protein-level interactions. There remains a lack of literature that systematically positions “FKBP5-DNAm” as a central and reversible epigenetic modifier and comparatively examines its roles across psychiatric, metabolic, and oncological settings. The potential of FKBP5-DNAm to serve as a cross-disease node linking psychological stress, physiological homeostasis, and malignant transformation remains underexplored. This review aims to establish a unified “epigenetic regulatory framework” centered on FKBP5-DNAm (Figure 1). It delineates both conserved and disease-specific methylation patterns across regulatory regions-such as promoters and intronic enhancers-and examines their downstream effects on GR signaling, cellular stress, and inflammatory pathways including NF-κB. Beyond synthesizing mechanistic insights, we also evaluate the translational potential of FKBP5-DNAm as a biomarker and explore intervention strategies-ranging from behavioral and environmental approaches to advanced epigenetic editing-highlighting both opportunities and challenges in clinical application. By integrating evidence from psychiatry, metabolism, and oncology, this review seeks to elucidate how FKBP5-DNAm functions as a molecular link between environmental exposure, physiological dysfunction, and disease pathogenesis, thereby fostering interdisciplinary dialogue and advancing FKBP5 research from bench to clinical application.

## 2 Content

### 2.1 Overview of FKBP5 gene and its biological functions

#### 2.1.1 FKBP5 gene structure and expression regulation

FKBP5, a gene originally cloned from a HeLa cancer cell cDNA library, is located in the 6p21.31 region of human chromosome 6, spanning approximately 156 kb (Storer et al., 2011; Hähle et al., 2019). The gene’s complex structure is regulated by multiple factors, including its own sequence variation and epigenetic modifications—notably, DNAm at multiple promoter regions. The FKBP5 gene encodes FKBP5 protein, and its key functional domains define its activity. The N-terminus contains two FK506-binding domains (FKBD1 and FKBD2), which confer peptidyl-prolyl cis-trans isomerase activity and mediate interaction with immunosuppressants. Central to its cochaperone function is a tetratricopeptide repeat (TPR) domain, essential for binding heat shock protein 90 (Hsp90) and incorporation into the GR molecular chaperone complex. While the TPR domain also mediates interactions with proteins such as WD-repeat protein interacting with phosphoinositides (WIPI), linking FKBP5 to autophagic pathways, this represents a secondary function relative to its primary role in GR regulation. A C-terminal domain contributes to protein stability and subcellular localization (Balsevich et al., 2017; Zannas et al., 2019; Engelhardt et al., 2021; Qiu et al., 2022).

The transcriptional activation of FKBP5 is mediated by the direct binding of the ligand-activated GR to multiple enhancer-like glucocorticoid response elements (GREs) distributed across its gene locus, most notably within introns 2, 5, and 7. GR binding to this cluster of GREs initiates robust, dose- and time-dependent transcription of FKBP5 mRNA upon GC exposure or psychological stress. This multi-GRE architecture facilitates sensitive and strong upregulation of FKBP5, which then functions as a critical intracellular feedback regulator of the GC receptor signaling pathway (Klengel et al., 2013; Anti et al., 2025; Kim et al., 2025). Clinical studies involving prenatal intervention indicate that dexamethasone (DEX) treatment crosses the placental barrier and activates GRs in the developing fetal brain (Van’t Westeinde et al., 2020). This exposure can disrupt key neurodevelopmental processes, ultimately influencing long-term brain structure. Research consistently identifies FKBP5 as a central and highly responsive component of the GC signaling pathway. *In vitro* models of neural differentiation confirm that acute DEX stimulation potently upregulates FKBP5 expression in a dose- and time-dependent manner, underscoring its role as a core stress-responsive gene (Babaniyi et al., 2023). The regulation of FKBP5 involves a well-characterized epigenetic mechanism. DNAm levels within the FKBP5 gene typically show a negative correlation with its expression; lower methylation is associated with higher FKBP5 mRNA and protein levels (Mendonça et al., 2021; Choi et al., 2021; Cox et al., 2021). This epigenetic state is clinically significant, as it is linked to an increased susceptibility to stress-related psychiatric disorders. In metabolic disorders such as obesity and insulin resistance, epigenetic analyses of patient blood samples have identified reduced DNAm at specific regulatory regions of the FKBP5 gene (Kang et al., 2020; Willmer et al., 2022). This hypomethylation is associated with increased FKBP5 expression, which impairs GR feedback and dysregulates the HPA axis. The resulting aberrant GC signaling enhances processes like hepatic gluconeogenesis and lipolysis, thereby exacerbating metabolic dysfunction. A similar mechanism linking early-life stress, FKBP5 hypomethylation, and subsequent disease risk is observed in psychiatric disorders (Karlbauer et al., 2024).

The functional single-nucleotide polymorphism (SNP) rs1360780 is located within intron two of the FKBP5 gene, approximately 50 kb downstream of the transcriptional start site (TSS). Its minor (T) allele resides in an enhancer region that harbors GREs (Stramecki et al., 2021; Rao et al., 2016; Arancibia et al., 2025). The presence of the T allele is associated with critical chromatin structural modifications that potentiate transcription. Firstly, the risk allele facilitates the recruitment of the TATA-box binding protein, promoting direct chromatin looping between intron two and the gene promoter. Secondly, upon exposure to childhood adversity, T-allele carriers exhibit significant DNA demethylation at these intronic GREs. These two mechanisms act synergistically to robustly enhance the functional communication between the enhancer and the promoter, leading to markedly increased FKBP5 mRNA expression following GR activation. This sustained overexpression is mechanistically linked to impaired GR signaling and reduced hormone sensitivity (Binder et al., 2004; Paakinaho et al., 2010; Klengel et al., 2013). Beyond DNAm, FKBP5 expression is modulated by non-coding RNAs, adding another layer of regulatory complexity. For example, miR-365-3p can repress FKBP5 by binding to its 3′untranslated region (UTR), influencing cellular metabolism (Chen H. et al., 2024). Conversely, circular RNA circ-FKBP5 can promote osteogenic differentiation by sequestering miR-205-5p (Shen et al., 2023). These findings illustrate the multi-level regulation of FKBP5 across different biological contexts. FKBP5 expression is also regulated at the level of chromatin structure and histone modifications, showed that GC signaling induces rapid chromatin remodeling and histone acetylation at the FKBP5 locus to facilitate its transcriptional activation (Paakinaho et al., 2010).

In summary, FKBP5 expression is governed by a multifaceted regulatory network involving genetic variation, DNAm, and SNP, etc. This review focuses on DNAm as a central epigenetic mechanism regulating FKBP5, underscoring its importance in understanding disease mechanisms and identifying potential therapeutic targets.

#### 2.1.2 Function of FKBP5 protein and its role in cellular signaling pathways

The FKBP5 protein, encoded by the FKBP5 gene, has a molecular weight of 51 kDa and is also referred to as FKBP51 protein. This protein participates in regulating key cellular signaling pathways. The GR is constitutively sequestered in a multi-chaperone complex, primarily comprising Hsp90, Hsp70, and immunophilins such as FKBP5 protein (Pratt and Toft, 1997; Binder, 2009). In its unliganded state, this complex maintains the GR in a high-affinity conformation, poised for hormone binding while preventing its nuclear translocation. Upon passive diffusion across the plasma membrane, GCs bind to the GR, inducing a conformational change that triggers its dissociation from the chaperone complex and subsequent translocation into the nucleus, where it acts as a transcription factor. By integrating into this complex, FKBP5 protein reduces the GR’s affinity for cortisol, impedes its nuclear translocation, and thereby attenuates further GC signaling. This establishes an ultra-short negative feedback loop that restrains excessive activation of the stress response. Dysregulation of this feedback mechanism-often driven by epigenetic alterations such as FKBP5 hypomethylation and consequent overexpression-constitutes a critical factor in the development of GC resistance and increased susceptibility to stress-related pathologies (Klengel et al., 2013). This epigenetic modulation of FKBP5 significantly influences GC signaling, thereby affecting stress response and endocrine homeostasis, particularly within the central nervous system (Liu et al., 2025).

Beyond its canonical role in the GR pathway, FKBP5 protein exhibits pleiotropic functions influencing processes ranging from cell differentiation and apoptosis to metabolism and psychiatric disorders. Mitochondria, central to energy homeostasis, stress response, cell cycle regulation, and redox balance, represent a key site for FKBP5 protein action. A studies using pancreatic beta-cell models demonstrate that elevated FKBP5 protein expression promotes cellular apoptosis under inflammatory conditions. Conversely, inhibiting FKBP5 protein enhances beta-cell viability through the AKT/FOXO1 signaling pathway, identifying FKBP5 protein as a contributor to diabetic pathophysiology and a potential therapeutic target (Liu et al., 2023). FKBP5 protein modulates transcription by competing with CDK9, thereby inhibiting the positive transcription elongation factor b complex and reducing RNA polymerase II phosphorylation. This leads to global impairment of transcriptional elongation and suppression of proliferation-related genes, suggesting a role in stress-induced transcriptional reprogramming (Mall et al., 2022). FKBP5 protein also regulates autophagy through direct interaction with WIPI protein via its TPR domain, influencing autophagosome formation and flux progression (Bajaj et al., 2022). In immune regulation, FKBP5 protein inhibits virus-induced interferon production by modulating transcription factors IRF3 and IRF7. Studies in zebrafish demonstrate that FKBP5 protein promotes degradation of these factors via selective autophagy, highlighting its role as a negative regulator of antiviral responses (Wang Y. et al., 2025). Additionally, in metabolic contexts, FKBP5 protein overexpression drives abnormal adipocyte proliferation through activation of the mTORC1 pathway. This is supported by single-cell RNA sequencing of patient-derived cells, where mTORC1 inhibition alleviated disease phenotypes (Chen M. et al., 2024). These findings collectively position FKBP5 protein as a multifunctional regulator influencing transcription, autophagy, immunity, and metabolism across various physiological and pathological contexts. The protein localizes to mitochondria through its TPR domain, where it exerts an anti-apoptotic effect. Notably, FKBP5 protein exhibits dynamic subcellular redistribution: upon cellular differentiation or stress onset, it translocates from mitochondria to the nucleus. Nuclear FKBP5 protein enhances telomerase enzymatic activity. This mitochondrial-nuclear shuttling is reversible, allowing FKBP5 protein to re-accumulate in mitochondria under specific conditions, facilitating a functional switch that contributes to cellular adaptation (Zgajnar et al., 2024).

FKBP5 protein augments inflammatory responses and modulates apoptosis through activation of the NF-κB signaling pathway in diverse pathological contexts. In kidney stone formation, FKBP5 protein promotes crystal adhesion, apoptosis, and pro-inflammatory M1 macrophage polarization via NF-κB (p65) activation, accelerating renal lithogenesis (Song et al., 2023). Similarly, in neuropathic pain, peripheral nerve injury induces FKBP5 protein upregulation and NF-κB activation in the spinal dorsal horn. FKBP5 protein and NF-κB form a positive feedback loop that amplifies pro-inflammatory cytokine production, sustaining chronic pain states (Wang X. et al., 2025). Detecting FKBP5 protein expression may thus help predict susceptibility to chronic pain and guide early intervention. FKBP5 also mediates neuroinflammation by modulating microglial activation. In experimental models, FKBP5 deficiency attenuates microglial activation and reduces pro-inflammatory gene expression following immune challenge, indicating its role in facilitating neuroinflammation through NF-κB (Gan et al., 2024). Collectively, these studies identify FKBP5 protein as a key amplifier of NF-κB-driven inflammation across renal, pain, and neuroimmune disorders, highlighting its potential as a therapeutic target for suppressing maladaptive inflammation.

In summary, FKBP5 protein functions as a signaling regulator and molecular scaffold that not only modulates GR activity but also broadly influences stress response, immune function, autophagy, and inflammatory signaling. Its multifunctional roles involve key pathways such as AKT, NF-κB, and AMPK/mTOR, contributing to the pathogenesis of diverse diseases and underscoring its biological significance and clinical potential.

### 2.2 The regulatory mechanism of DNAm in FKBP5 gene

#### 2.2.1 The basic principles of DNAm and its impact on gene expression

DNAm is an epigenetic modification involving the addition of a methyl group to the 5-carbon position of cytosine residues in DNA, forming 5-methylcytosine. This process predominantly occurs in CpG islands—genomic regions enriched in cytosine–guanine dinucleotide sequences—often located near gene promoters. DNAm regulates gene expression by modulating transcription factor binding and chromatin structure, thereby exerting profound effects on transcriptional activity (Dhar et al., 2021; Cortés-Mancera et al., 2022). Methylation status critically determines gene activity. High promoter methylation generally correlates with transcriptional silencing, as it impedes transcription factor binding and recruits methyl-CpG-binding proteins such as MeCP2 and associated repressor complexes. These interactions promote a condensed heterochromatin state that restricts transcriptional access (Ma et al., 2020). In contrast, low promoter methylation typically supports active gene expression (Liu T. et al., 2024). An exploratory study using Illumina Infinium MethylationEPIC BeadChip technology reveals that postmortem brain tissue from individuals who died by suicide shows high methylation in promoter or enhancer regions, correlating with transcriptional repression. Expression levels of 135 genes, including FKBP5, are significantly negatively correlated with their DNAm levels (Cabrera-Mendoza et al., 2020). Both global hypomethylation of CpG islands in FKBP5 and demethylation at specific CpG sites activate its transcription (Piyasena et al., 2016; Misiak et al., 2020). Additionally, gene body methylation contributes to transcriptional stability and regulates RNA splicing (Jia et al., 2025). FKBP5-DNAm exhibits notable tissue specificity. Animal models reveal distinct DNAm profiles across brain regions—such as the PFC and hippocampus—and peripheral blood, which may reflect tissue-specific responses to HPA axis activation under stress (Yusupov et al., 2024).

Abnormal FKBP5-DNAm leads to its dysregulated expression, representing a core epigenetic mechanism influencing susceptibility to mental illness. Supporting this, a multimodal study combining methylation analysis and neuroimaging in adults demonstrated that lower FKBP5-DNAm in a stress-sensitive intronic region correlates with enhanced connectivity in brain networks involved in self-referential and emotional processing (Kremer et al., 2024). Early-life environmental insults can induce persistent epigenetic alterations. In a mouse model, alcohol exposure during a critical developmental period caused promoter hypomethylation and increased FKBP5 protein expression in the adult PFC, which was associated with heightened anxiety-like behavior (Dursun et al., 2025). Similarly, in humans, children of depressed mothers exhibited reduced FKBP5-DNAm and corresponding gene upregulation, which predicted HPA axis hypersensitivity and increased depression risk, suggesting a transgenerational transmission of vulnerability via epigenetic mechanisms (Mendonça et al., 2023). This link extends beyond psychiatric disorders. A systematic review synthesized that childhood trauma induces long-term FKBP5 hypomethylation, which epigenetically embeds early adversity into biological systems. This programming elevates the risk not only for mental illness but also for cardiometabolic disorders, indicating FKBP5’s role as a molecular nexus connecting psychological stress with systemic physiological dysregulation (Womersley et al., 2022).

In summary, interactions between genetic polymorphisms and environmental stress shape FKBP5-DNAm patterns, driving dysregulated gene expression and elevating susceptibility to psychiatric disorders such as depression and PTSD.

#### 2.2.2 Progress in FKBP5-DNAm detection technology

The detection of FKBP5-DNAm gene employs several techniques, including methylation-specific PCR (MSP), bisulfite sequencing PCR (BSP), pyrosequencing (which utilizes bisulfite-converted, PCR-amplified DNA), high-throughput methylation arrays, and emerging single-cell methylation sequencing. These methods characterize key functional regions and dynamic changes in FKBP5-DNAm.

MSP determines the methylation status of specific CpG sites using primers designed for methylated and unmethylated sequences after bisulfite conversion of DNA. This technique offers high sensitivity but lacks quantitative capability, making it suitable for monitoring methylation in regulatory elements such as intron 7, which is associated with mental disorders (Beach et al., 2022; Seo et al., 2023). BSP converts unmethylated cytosine (C) to uracil (U) while retaining methylated cytosine unchanged. When combined with high-throughput sequencing, BSP provides single-nucleotide resolution methylation analysis with high accuracy and quantitative capacity, enabling detailed mapping of CpG methylation patterns across the FKBP5 gene. High-precision targeted BSP methods, such as HAM-TBS, have been used in FKBP5 research to compare methylation patterns across tissues (e.g., blood and brain regions) in both mouse models and humans, and to assess responses to stress (Yusupov et al., 2023). Pyrosequencing involves amplifying a single-stranded DNA template, hybridizing it with primers, and sequentially adding deoxyribonucleotides. Complementary base incorporation releases pyrophosphate (PPi), which generates a detectable light signal. This method enables real-time, high-resolution sequencing with rapid turnaround and high-throughput quantitative capability, allowing base-by-base DNA analysis (Higashimoto et al., 2023; Muehlhan et al., 2020). High-throughput methylation arrays, such as Illumina’s 450K and EPIC 850K chips, simultaneously assess methylation at hundreds of thousands to millions of CpG sites genome-wide. These tools are widely used in large-scale epigenomic studies to identify differential methylation sites in FKBP5 under various disease conditions—including cancer and psychiatric disorders—thus aiding in elucidating disease mechanisms and discovering potential biomarkers (Titanji et al., 2022; Cugliari et al., 2021). Recently, single-cell BSP has enabled DNAm analysis at single-cell resolution, revealing cellular heterogeneity and enhancing understanding of spatiotemporal dynamics in epigenetic regulation (Wang and Li, 2024). This technique shows great promise for examining FKBP5-DNAm in neurological and other diseases and may provide valuable insights into tumor heterogeneity and immune microenvironment regulation.

In summary, FKBP5-DNAm detection has evolved from traditional qualitative PCR to high-throughput quantitative arrays and sequencing technologies. These advances provide robust tools for probing the epigenetic regulation of FKBP5 in cancer and other diseases.

#### 2.2.3 FKBP5 gene specific methylation sites and their regulatory characteristics

At the regulatory level, FKBP5-DNAm occurs primarily within functional elements such as promoters, enhancers, and CTCF-binding sites, where it modulates chromatin structure and transcription factor accessibility (Wang et al., 2023). In disease contexts, promoter hypomethylation is frequently associated with transcriptional upregulation. For example, in dilated cardiomyopathy, reduced methylation in the FKBP5 promoter correlates with elevated mRNA levels and inflammatory cardiac remodeling (Wada et al., 2021). Similarly, in adrenal insufficiency, average methylation across 54 promoter CpGs inversely correlates with GC replacement dose, reflecting endocrine-dependent regulation (Chifu et al., 2025). Specific CpG sites, such as cg22363520 and cg00862770 in intron 7, show methylation changes linked to psychiatric and metabolic disorders. Exercise can modulate methylation at these sites, with distinct patterns observed between diabetic and non-diabetic individuals (Yang et al., 2025). Moreover, the SNP rs1360780 interacts with early trauma to demethylate intron 7 CpGs, increasing FKBP5 expression and contributing to HPA axis dysregulation in psychiatric conditions (Mihaljevic et al., 2021). Clinically, methylation at site cg07485685 has been identified as a predictor of PTSD symptom severity and resilience capacity (Miller et al., 2020). Collectively, these findings underscore that FKBP5-DNAm at specific regulatory CpGs serves as a dynamic interface between genetic variation, environmental exposure, and disease susceptibility across psychiatric, metabolic, and stress-related disorders.

In summary, FKBP5-DNAm occurs predominantly in promoter and intronic regions and generally correlates negatively with gene expression. This methylation exhibits high site- and tissue-specificity and is influenced by genetic SNP and environmental factors such as childhood trauma, alcohol use, and exercise, reflecting a complex epigenetic regulatory landscape. Investigating these key methylation sites not only elucidates FKBP5-related disease mechanisms but also supports the development of methylation-based predictive and therapeutic strategies.

#### 2.2.4 The influence of environmental factors on FKBP5-DNAm

DNAm status of the FKBP5 gene is regulated by various environmental factors, including lifestyle elements such as stress, smoking, alcohol consumption, and medication use, as well as socioeconomic status. These external factors influence FKBP5 expression by altering its DNAm levels, forming a critical link in the pathogenesis of multiple diseases.

Emerging research utilizing integrated epigenetic, neuroimaging, and behavioral methods demonstrates that FKBP5-DNAm is linked to functional variations in prefrontal-limbic brain circuits and modulates individual differences in emotional reactivity to daily stressors in humans (Zhen-Duan et al., 2025). Beyond human studies, evidence from free-living animal models confirms that social experiences directly shape epigenetic states. Exposure to aggressive social signals rapidly induces dynamic methylation changes in FKBP5 homologs, establishing a direct causal pathway from social stress to epigenetic alteration in wild species (McNew et al., 2024). Furthermore, early life adversity exerts intergenerational effects through epigenetic synchronization. In mother-infant pairs, maternal early adversity shapes both maternal and offspring methylation profiles at birth. Continued cohabitation further synchronizes their epigenomes, increasing offspring disease susceptibility and revealing epigenetic similarity as a mechanism for the intergenerational transmission of environmental effects (Labaut et al., 2024). Maternal psychological trauma during pregnancy can become biologically embedded in offspring through epigenetic mechanisms. A prospective cohort study demonstrated that prenatal post-traumatic stress symptoms are associated with reduced FKBP5-DNAm. This hypomethylation leads to increased FKBP5 protein expression, impaired GC signaling, and HPA axis dysfunction, providing a molecular basis for the intergenerational transmission of trauma and health disparities (Stroud et al., 2024). Furthermore, prenatal psychological distress—including anxiety, depression, and stress—alters the placental methylation landscape of GC pathway genes, with FKBP5 being a key target. These epigenetic modifications correlate with measurable neonatal growth outcomes, such as birth weight and length, establishing a clear pathway from “maternal psychological state-placental epigenetic programming -offspring developmental outcomes.” These findings offer precise molecular targets for early intervention strategies aimed at breaking the cycle of intergenerational trauma (Castro-Quintas et al., 2024).

In summary, environmental factors contribute to disease by modulating FKBP5-DNAm, thereby influencing its expression and function. The plasticity of FKBP5-DNAm reflects dynamic gene–environment interactions and opens new avenues for epigenetics-based disease prevention and intervention. Future research should combine large-scale longitudinal and transgenerational studies with precise environmental exposure assessments to further elucidate mechanisms and clinical implications of environmental effects on FKBP5-DNAm.

### 2.3 Study on the association between FKBP5-DNAm and mental disorders

#### 2.3.1 Changes in FKBP5-DNAm in depression and anxiety disorders

**FIGURE 2:**
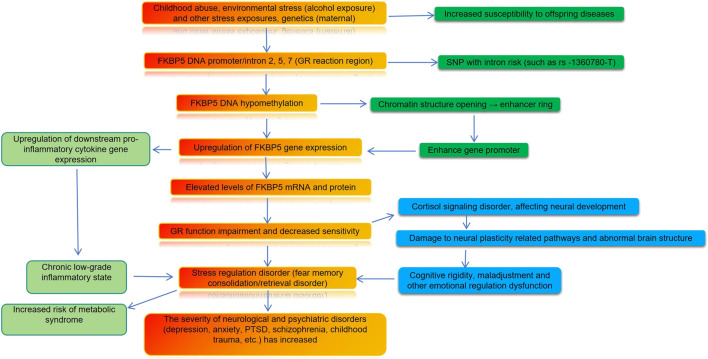
FKBP5 methylation and its relevance in psychiatric disorders.

Early research established FKBP5’s role in psychiatric and neurological disorders through its interaction with the GR and HPA axis (Figure 2). In alcohol use disorder, the rs1360780 SNP (T allele) in FKBP5 correlates with higher psychological resilience, suggesting genetic influences on stress adaptation (Park et al., 2021). Clinical studies consistently report altered FKBP5 promoter DNAm in psychiatric disorders, though the direction of change shows variability. Research in adults with HIV—a population facing significant psychosocial stress—demonstrates that high FKBP5-DNAm suppresses its expression and enhances GR signaling, promoting HPA axis recovery. Conversely, low methylation correlates with more severe clinical symptoms. Trauma exposure associates with distinct methylation patterns, while positive coping strategies may buffer these epigenetic alterations, supporting a biopsychosocial model of stress regulation (Tomlinson et al., 2025). Notably, a large study of healthy women found no significant association between stressors and DNAm in HPA axis-related genes after multiple testing correction, suggesting that methylation patterns are influenced by experiences across the lifespan rather than early exposures alone (Shields et al., 2021). FKBP5-DNAm shows clinical relevance as a potential biomarker. In depression and anxiety, lower methylation often associates with more severe symptoms yet may predict better response to certain antidepressants, indicating utility for treatment personalization (Alahmad et al., 2025). Objective measures of HPA axis function, such as the dexamethasone suppression test, correlate with methylation changes in stress-related genes including FKBP5 and NR3C1. These epigenetic alterations are enriched in pathways involving nervous system development and cell signaling, ultimately influencing emotional regulation and cognitive function (Chatzittofis et al., 2021). The connection between early trauma and psychopathology is further elucidated through epigenetic mechanisms. In borderline personality disorder, severe childhood abuse associates with reduced FKBP5-DNAm in intron 7, enhancing FKBP5 expression and impairing HPA axis negative feedback. These methylation changes partially mediate the relationship between early abuse and clinical symptoms, contributing to the disorder’s etiology (Flasbeck and Brüne, 2021).

Overall, altered FKBP5-DNAm plays a significant role in the pathogenesis of mental disorders such as depression and anxiety. Further investigation into its epigenetic regulatory mechanisms may clarify disease progression and support the development of personalized treatment strategies.

#### 2.3.2 Epigenetic regulation in PTSD

Aberrant FKBP5-DNAm is a key epigenetic mechanism in PTSD, influencing disease risk, symptom severity, and treatment response. Research utilizing genetic models demonstrates that the high-risk T allele at SNP rs1360780 is associated with reduced FKBP5-DNAm, increased chromatin accessibility, and subsequent gene upregulation upon stress exposure. This impairs GR feedback and HPA axis regulation, explaining the heightened vulnerability of risk allele carriers to PTSD (Nold et al., 2021; Arancibia et al., 2025). Critically, FKBP5-DNAm patterns are shaped by gene-environment interactions, particularly with childhood trauma. Individuals carrying the risk genotype who experience early-life adversity exhibit significant demethylation in regulatory regions like intron 7, leading to FKBP5 overexpression and more severe PTSD symptoms after adult trauma. This underscores the biological embedding of trauma and provides mechanistic insight relevant to clinical and forensic assessments (Arancibia et al., 2025; Nöthling et al., 2025). The field is rapidly evolving from basic discovery toward translational research, with FKBP5-DNAm recognized as a current focal point (Nie et al., 2022). Systematic reviews consolidate that low FKBP5-DNAm enhances GR inhibition and elevates PTSD risk, noting these epigenetic marks are dynamic rather than fixed (Khan et al., 2025). Furthermore, methylation shows significant heterogeneity across individuals, influenced by trauma type, genetic background, sex, and treatment (Appleton, 2025). Maternal childhood adversity, especially when coupled with PTSD, is associated with distinct FKBP5-DNAm patterns in both mothers and their newborns, indicating that epigenetic mechanisms may transmit vulnerability across generations (Grasso et al., 2020).

In summary, FKBP5-DNAm contributes to elucidating PTSD pathogenesis and holds clinical promise as a diagnostic and prognostic biomarker. Future studies should validate its stability and specificity across diverse populations and explore its potential for individualized treatment strategies.

### 2.4 The role of FKBP5-DNAm in malignant tumors and other diseases

#### 2.4.1 Abnormal methylation of FKBP5 in malignant tumors and its mechanism

**FIGURE 3:**
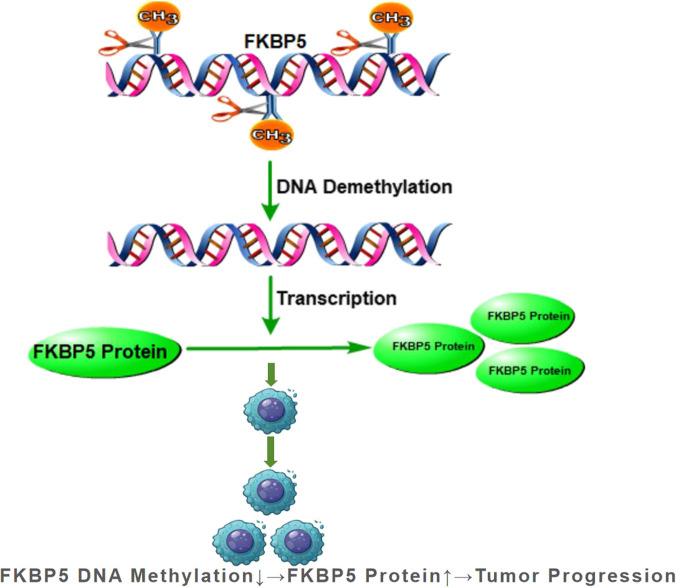
Schematic diagram of the mechanism of FKBP5 DNA methylation abnormalities in different cancers.

Emerging evidence implicates FKBP5 as a significant player in tumorigenesis, progression, and prognosis across various malignancies, though its precise mechanisms and clinical utility remain areas of active investigation. In colorectal cancer (CRC), FKBP5 exerts an oncogenic role by activating the NF-κB signaling pathway, promoting tumor cell proliferation, migration, and invasion. The immunosuppressant FK506 demonstrates therapeutic efficacy in preclinical models, positioning FKBP5 as both a prognostic marker and a potential therapeutic target (Liu W. et al., 2024). Epigenetic regulation of FKBP5 significantly influences cancer outcomes (Figure 3). In malignant pleural mesothelioma, low methylation at a specific CpG site in the 5′UTR upregulates FKBP5 protein expression and predicts reduced patient survival more effectively than conventional inflammatory markers (Cugliari et al., 2020). Similarly, in metastatic castration-resistant prostate cancer, FKBP5 expression is governed by super enhancer elements. Inhibition of this transcriptional activity effectively suppresses tumor growth, revealing a promising therapeutic avenue (Zeng et al., 2023). Beyond direct oncogenic effects, FKBP5-DNAm status modulates tumor-immune interactions. In early-stage lung adenocarcinoma, FKBP5 has been incorporated into integrated prognostic models that combine methylation and immune signatures. Aberrant FKBP5-DNAm correlates with altered CD8^+^ T-cell infiltration and immune escape mechanisms, highlighting its role in shaping the tumor immune microenvironment (Ren et al., 2021). These collective findings underscore FKBP5’s dual significance as both a methylation-driven regulator of tumor biology and a promising target for therapeutic intervention across multiple cancer types.

Overall, dysregulated FKBP5 DNAm directly influences its transcriptional activity and contributes to tumorigenesis through signaling pathway alteration and modulation of the tumor microenvironment (Table 1). As a molecular phenotype, FKBP5-DNAm status holds promise for clinical diagnosis and prognosis, offering novel targets and strategies for precision oncology.

**TABLE 1:** Cross-cancer comparison of FKBP5 methylation patterns and clinical translation strategies.

Cancer type	FKBP5 protein expression	Methylation status	Key regulatory mechanism	Clinical translation strategy
Malignant pleural mesothelioma	Upregulated	Hypomethylation at specific 5′UTR sites	Interaction with inflammatoryfactors	Prognostic biomarker superior to CRP
Prostate cancer	Upregulated	Super-enhancer driven	Activation of androgen signaling pathway	Potential strategy: targeting SE-associated elements
Lung adenocarcinoma	Variable	Region-specific differential methylation	Modulation of immune infiltration (CD8+ T cells)	Combined immunoscore modeling for clinical stratification

#### 2.4.2 Epigenetic regulation in metabolic and endocrine diseases

FKBP5-DNAm plays a significant role in metabolic diseases such as type 2 diabetes and obesity, though its regulation and functional impact differ notably from its role in psychiatric or oncological contexts. In diabetes and obesity, key CpG sites including cg22363520 and cg00862770 show elevated methylation. Exercise exerts site-specific demethylation effects: at cg22363520 in non-diabetic individuals and at cg00862770 in diabetic patients, thereby improving insulin sensitivity and metabolic function (Yang et al., 2025). A distinct methylation profile is observed in obesity, where increased methylation at specific FKBP5 CpGs in adipose tissue correlates with higher BMI, insulin resistance, and systemic inflammation. Notably, methylation in gluteal adipose tissue corresponds to reduced FKBP5 mRNA, suggesting a tissue-specific regulatory mechanism (Willmer et al., 2020). FKBP5-DNAm is also implicated in diabetic complications. In end-stage kidney disease secondary to type 1 diabetes, differential methylation is detected at FKBP5-related CpG sites, highlighting its involvement in renal pathology through pathways such as TGF signaling and Th17 differentiation (Smyth et al., 2021). Similarly, in endocrine disorders, patients with Cushing’s syndrome exhibit significant FKBP5 hypomethylation in blood cells, reflecting GC excess (Armignacco et al., 2022). These findings underscore that FKBP5-DNAm is not only a biomarker of metabolic dysfunction but also a dynamic and reversible regulatory mechanism, offering potential targets for therapeutic interventions in metabolic and endocrine diseases.

In summary, future research should continue to investigate epigenetic mechanisms, integrating genetic and environmental factors to advance precision medicine in metabolic and endocrine diseases.

#### 2.4.3 FKBP5-DNAm in inflammatory and autoimmune diseases

FKBP5 plays a pivotal role in inflammatory diseases and immune regulation, where its aberrant DNAm serves as a key pathogenic mechanism. In ulcerative colitis (UC), elevated FKBP5 expression links chronic inflammation to subsequent oncogenic risk in the colon (Xia et al., 2021). Similarly, in dilated cardiomyopathy (DCM), hypomethylation of the FKBP5 promoter in peripheral blood leukocytes correlates with increased FKBP5 and IL-1β expression, driving inflammatory cardiac remodeling (Wada et al., 2021). Chronic stress induces systemic epigenetic reprogramming that bridges neuroendocrine and immune dysfunction. In middle-aged women under prolonged stress, FKBP5 hypomethylation occurs alongside HPA axis dysregulation, suggesting an integrated mechanism for heightened disease vulnerability (Palma-Gudiel et al., 2020). Exposure to synthetic GC during pregnancy also reduces FKBP5-DNAm in placental enhancer regions, elevating expression of inflammatory gene modules associated with preeclampsia and potentially affecting fetal immune development (Czamara et al., 2021). In specific immune-mediated diseases, FKBP5-DNAm is altered by environmental exposures. Smoking-associated methylation changes in FKBP5 contribute to increased Crohn’s disease risk (Wang et al., 2022). In alcohol-related liver disease (ALD), ethanol induces FKBP5 promoter hypomethylation, leading to upregulated FKBP5 protein expression. This activates the Hippo pathway, resulting in YAP/TEAD1-driven transcription of pro-inflammatory chemokines like CXCL1 and promoting hepatic inflammation. Notably, FKBP5 deletion attenuates ALD, highlighting its therapeutic potential (Kusumanchi et al., 2021). These findings are consistent with broader epigenetic dysregulation, including DNAm and chromatin remodeling, observed in inflammatory conditions (Habash et al., 2022).

In summary, aberrant DNAm of FKBP5 regulates its expression, modulates immune cell function, amplifies inflammatory responses, and contributes to the pathology of various inflammatory and autoimmune diseases. Future studies should further clarify the specific regulatory roles of FKBP5-DNAm in different inflammatory contexts and evaluate its clinical applicability. The accumulated evidence demonstrates the significant potential of FKBP5-DNAm status in early diagnosis and prognostic assessment across psychiatric, malignant, endocrine, and immune-related disorders.

### 2.5 The clinical application potential and therapeutic target value of FKBP5-DNAm

FKBP5-DNAm is increasingly recognized as a promising biomarker for early diagnosis, prognostic assessment, and monitoring therapeutic response across neuropsychiatric disorders. In adolescents, positive environmental interventions-such as family support and psychotherapy-an reverse adverse FKBP5-DNAm patterns linked to genetic risk and early trauma, improving emotional outcomes and offering optimism for personalized depression treatment (Guo et al., 2021). Notably, methylation patterns in key regulatory regions of FKBP5, particularly in intron 7, show high consistency between peripheral blood and brain tissues (e.g., PFC and hippocampus). This cross-tissue correlation supports the use of blood-based FKBP5-DNAm as a reliable and accessible biomarker for molecular subtyping and diagnostic support in clinical cohorts (Yusupov et al., 2024). In PTSD, altered HPA axis function-including enhanced negative feedback and reduced cortisol levels-represents a core feature. Genetic and epigenetic variations in FKBP5 and NR3C1 correlate with hypocortisolism, and epigenetic changes parallel symptom improvement during trauma-focused psychotherapy (Fischer et al., 2021). Further reinforcing this, an 8-week mindfulness-based intervention in veterans with PTSD induced dynamic changes in FKBP5 intron 7 methylation. These changes correlated positively with symptom reduction, highlighting the gene-epigenetic-environment interplay in response to non-pharmacological therapy (Lee et al., 2024). Collectively, these findings underscore the translational value of FKBP5-DNAm as a dynamic biomarker that reflects both disease vulnerability and therapeutic response, paving the way for epigenetically-informed clinical strategies.

FKBP5 inhibitors are widely regarded as a promising therapeutic strategy for depression, PTSD, and type 2 diabetes, with several candidate compounds showing positive results in preclinical studies (Agam et al., 2024). The immunosuppressant FK506 (tacrolimus), which targets FKBP5 protein, is widely used in clinical therapy. It demonstrates efficacy in treating hormone-refractory or fulminant UC. As a known precursor to CRC, UC responds well to FK506, supporting further investigation into inflammation–cancer transition mechanisms (Fuentes-Valenzuela et al., 2025). It remains unclear whether FK506 can partially reverse DNAm at specific sites or modulate methylation levels across CpG islands by inhibiting FKBP5 expression. However, this direction holds significant research potential. A significant advancement in the targeted pharmacology of FKBP5 protein, distinct from epigenetic modulators, is the development of SAFit2 (Selective Antagonist of FKBP5 by induced fit, second generation). SAFit2 is a highly specific and potent small-molecule inhibitor rationally designed to occupy the FKBD1. This binding inhibits its peptidyl-prolyl isomerase activity and disrupts its critical interaction with the GR chaperone complex. The therapeutic relevance of SAFit2 is particularly pronounced in the context of methylation-associated pathophysiology. By directly antagonizing the overexpressed FKBP5 protein, SAFit2 can potentially break the vicious cycle driven by FKBP5 hypomethylation and alleviate the downstream pathological consequences of this aberrant epigenetic state (Cruz et al., 2023; Tian et al., 2024; Ejiohuo et al., 2025). In obesity, integrated bioinformatic analyses of public datasets reveal elevated FKBP5 expression alongside reduced levels of CD8^+^ T and natural killer cells. This immune-epigenetic signature helps identify potential therapeutic compounds, including atorvastatin, for repurposing in obesity management (Liu et al., 2021). Pharmacological modulation of DNAm offers a novel approach to alter disease trajectories, with FKBP5 representing a key candidate for such interventions (Smyth et al., 2021).

In addition to drug therapy, there are also gene editing strategies. The strategy utilizes a catalytically dead Cas9 (dCas9) protein fused to effector domains of DNA-modifying enzymes, such as the ten-eleven translocation dioxygenase for targeted demethylation or DNA methyltransferase (DNMT) for targeted methylation. Guided by sequence-specific single-guide RNAs (sgRNAs), these dCas9-effector constructs can be directed to specific CpG sites within regulatory regions to actively rewrite the local DNAm landscape (Liu et al., 2018; Marx et al., 2018; Liu et al., 2016). In a parallel and complementary therapeutic direction, conventional CRISPR/Cas9-mediated gene disruption (complete knockout) has been employed as a robust tool for functional validation. For instance, FKBP5 knockout via CRISPR/Cas9 in isolated human primary preadipocytes has been used to elucidate its role in adipogenesis and to investigate GC effects in human adipocytes, with the methodological feasibility concurrently validated using other genes (Kamble et al., 2020). Beyond above, emerging epigenetic editing technologies represent a precise strategy for targeting disease-associated DNAm patterns. Beyond above, locus-specific epigenetic editing tools are being developed to directly target FKBP5 regulation. Single-stranded methylated DNA probes designed for FKBP5 regulatory regions induce sustained, dose-dependent hypermethylation in human and mouse cell models. This targeted methylation attenuates GC-induced FKBP5 upregulation, providing a simple and safe alternative to CRISPR-based epigenome editing for potential treatment of stress-related psychiatric disorders (Modi et al., 2025). These approaches demonstrate the therapeutic potential of targeting FKBP5-DNAm, either through pharmacological modulation or direct epigenetic editing, opening new avenues for personalized medicine in metabolic and psychiatric conditions.

Given the multifaceted role of FKBP5 across disorders and its complex epigenetic regulation, current research increasingly focuses on combining epigenetic drugs with other therapies to achieve synergistic outcomes. However, not all interventions fully reverse established methylation patterns, as such plasticity operates within specific biological and contextual constraints. Moreover, clinical trials investigating FKBP5 methylation-targeted therapies remain limited, underscoring the need for more high-quality randomized controlled trials to evaluate their efficacy and safety.

## 3 Conclusion

DNAm of the FKBP5 gene plays a critical role in regulating its expression and function, attracting widespread attention in medical research. Studies demonstrate that aberrant FKBP5-DNAm is closely associated with psychiatric conditions such as depression and anxiety, as well as with cancers and metabolic diseases, underscoring the significance of epigenetic regulation in complex disorders. This perspective enhances understanding of disease etiology. Factors such as environmental stress, life events, and drug exposure influence FKBP5-DNAm status and expression levels, thereby affecting stress response pathways and disease vulnerability. However, discrepancies exist among studies regarding FKBP5-DNAm patterns and clinical relevance, likely due to variations in sample characteristics and detection methodologies. Future research should integrate multi-omics data across larger, multicenter cohorts to clarify these relationships. In precision medicine, FKBP5-DNAm testing enables early screening, risk stratification, and personalized treatment planning. In psychiatry, monitoring FKBP5-DNAm helps predict antidepressant response and guides personalized pharmacotherapy. In oncology, drug development targeting the FKBP5 pathway holds promise for improved treatment outcomes, enabling more accurate therapeutic matching while avoiding ineffective interventions.

In conclusion, the accumulating evidence solidifies the central role of dynamic FKBP5-DNAm as a key interface through which genetic predisposition and environmental exposures converge to influence disease risk and progression across psychiatric, metabolic, and neoplastic disorders. Its reversible nature further underscores its therapeutic potential. Future research should prioritize large-scale longitudinal studies to validate the temporal dynamics of FKBP5-DNAm in response to environmental and therapeutic interventions. The translation of these epigenetic insights into clinical practice holds promise for advancing personalized medicine, enabling more precise diagnostic stratification, prognostic evaluation, and targeted epigenetic therapies for a range of complex diseases.
